# Hæmaturia Simplex in a New-born Child

**Published:** 1888-05

**Authors:** Harold N. Moyer

**Affiliations:** Lecturer on Physiology, Rush Medical College; 434 West Adams Street


### HÆMATURIA SIMPLEX IN A NEW-BORN CHILD.

BY HAROLD N. MOYER, M. D.,
Lecturer on Physiology, Rush Medical College.

In the early part of January of the current year a child five days old came under my observation, suffering from hæmaturia. The child was small and extremely delicate, one of twins born after a tedious but not specially difficult labor. The first urine passed after birth contained a small quantity of blood; the next was much darker and stained the napkins like nearly pure blood. This continued until the fifth day, when I saw the child. The portions of the napkins stained with urine were of a uniform reddish-brown color, somewhat stiffened, but there was no evidence of clotting. Portions that were stained were cut out and macerated in water. The resulting fluid contained a large number of red blood corpuscles and some phosphates, but no crystals of uric acid or oxalate of lime. The fluid was markedly alkaline, but this might have been due to soap contained in the fabric. At no time was there any evidence of pain when the urine was passed. Temperature was normal, appetite good, bowels regular. No tenderness over hypogastric or lumbar region. On the evening of the fifth day a mild icterus developed. The skin was faintly yellow, the conjunctivæ were not colored, nor was there any evidence of inflammation about the umbilicus. The icteric attack faded on the second day, and was not associated with any disturbance of the general health. When the child was six days old the urine became clearer, and on the seventh day was free from blood. Since that time the child has continued perfectly well; it has grown stronger, and now presents the appearance of a healthy, well-nourished babe of three months.

So far as the literature of this subject is accessible to me, I have been able to find but little reference to the condition except the relation in which the symptom stands to cancer of the kidneys. This matter was carefully considered by A. Leibert (Jahrb. f. Khkde., 1884), who publishes a case of his own, two from Dr. Jacobi's practice, and gives a résumé of forty-eight published cases of cancer of the kidney occurring in children under eleven years of age. He found that hæmaturia was relatively more frequent in cancer of the kidneys in children than in grown people. In two-thirds of the cases it was the first symptom of the disease.

An examination of a large number of works on diseases of children, only elicits the uniform statement that hæmaturia is most marked in purpura scarlatina, croup, pneumonia, chronic exanthemata and cancer, tuberculosis, and cheesy degeneration of the kidneys. The condition which I have ventured to term "hæmaturia simplex" is nowhere referred to in any English work on diseases of children (with one exception), nor does it receive consideration in the ponderous German "handbook" (Handbuch der Kinderheilkunde). I do not, however, regard the symptom as a very rare one, though the literature of the subject is so meagre. A case is reported in the *Lancet* for 1880, the reference to which is not now accessible to me, and another is found in the work of Dr. Goodhart (A Guide to the Diseases of Children, by James Frederick Goodhart, edited by Louis Starr, M. D.). He says, after referring to hæmaturia in the graver conditions: "But besides all these, and more puzzling than they, children are brought to the out-patient room with a history of frequent passage of blood from the urine. Perhaps they are admitted, and the blood, present once or twice within the first few hours, disappears altogether, and does not reappear." The author then describes the case of a girl aged seven, who had suffered from hæmaturia for four months, not associated with pain or any disturbance of the general health, nor could the cause of the symptom be determined. When the patient was admitted the author remarked that "some of the features were those of vesical growth, but that it was a frequent hospital experience that children, with prolonged hæmaturia outside, speedily got well inside the hospital." Such proved to be the case with this little girl.

While this is the first case of hæmaturia in a young child that has ever come under my observation, yet, as I said before, I do not believe that the condition is very rare. The indications are to examine for any of the ordinary causes of hæmaturia, stone, renal or vesical, nephritis, or vesical growths. Unfortunately the malignant forms, cancer, tuberculosis, etc., can only be differentiated by time, but these later conditions are not to be inferred because the former are excluded.

I think, from what has already been said, that we are justified in adding the term "hæmaturia simplex" to the literature of pædiatrics, and that with advantage, as the practitioner when confronted with the array of deadly diseases with which the symptom is associated in most of our standard text-books, will make a fatal prognosis which will not be verified by the subsequent history.

434 West Adams Street.

